# Impact of Treatment on Systemic Immune-Inflammatory Index and Other Inflammatory Markers in Odontogenic Cervicofacial Phlegmon Cases: A Retrospective Study

**DOI:** 10.3390/biomedicines11061710

**Published:** 2023-06-14

**Authors:** Ciprian Ioan Roi, Alexandra Roi, Adrian Nicoară, Diana Nica, Laura Cristina Rusu, Andrada Soancă, Alexandru Cătălin Motofelea, Mircea Riviș

**Affiliations:** 1 Department of Anesthesiology and Oral Surgery, “Victor Babeș” University of Medicine and Pharmacy, Eftimie Murgu Sq. No. 2, 30041 Timișoara, Romania; ciprian.roi@umft.ro (C.I.R.); nicoara.adrian@umft.ro (A.N.); nica.diana@umft.ro (D.N.); rivis.mircea@umft.ro (M.R.); 2 Multidisciplinary Center for Research, Evaluation, Diagnosis and Therapies in Oral Medicine, “Victor Babeș” University of Medicine and Pharmacy, Eftimie Murgu Sq. No. 2, 30041 Timișoara, Romania; laura.rusu@umft.ro; 3 Department of Oral Pathology, “Victor Babeș” University of Medicine and Pharmacy, Eftimie Murgu Sq. No. 2, 30041 Timișoara, Romania; 4 Department of Periodontology, Faculty of Dental Medicine, Iuliu Hațieganu University of Medicine and Pharmacy, Victor Babes Street, No. 15, 40012 Cluj-Napoca, Romania; 5 Department of Internal Medicine, Faculty of Medicine, “Victor Babeș” University of Medicine and Pharmacy, 30041 Timișoara, Romania; alexandru.motofelea@umft.ro

**Keywords:** odontogenic infections, cervicofacial phlegmons, inflammatory markers, maxillofacial surgery, prognostic factors, systemic immune-inflammatory Index, NLR

## Abstract

Cervicofacial odontogenic infections can have an aggressive evolution with life-threatening complications. Management in many cases can be a challenge for clinicians, implying an extra focus on individual inflammatory parameters. The aim of this study is to evaluate the evolution of inflammatory markers for the included diagnosed odontogenic cervicofacial phlegmon cases at the moment of hospitalization and after receiving surgical and pharmaceutical treatment. Materials and methods: A total of 39 patients diagnosed with odontogenic cervicofacial phlegmons that were admitted to the Maxillofacial Surgery Department of the Emergency Hospital from Timisoara were included in the study. The main focus was the parameters represented by the systemic immune-inflammatory index (SII) based on neutrophil, platelet, and lymphocytes count; the neutrophil–lymphocyte ratio (NLR); C-reactive protein level (CRP); and white blood cell count (WBC) before and after the treatment as potential prognosis factors. Results: The results of the study after analyzing the included parameters revealed a significant difference between the calculated values of the SII, NLR, CRP, and WBC at admission and at time of discharge, being directly influenced by the treatment. Conclusions: SII, NLR, CRP, and WBC dynamic changes in severe cervicofacial odontogenic infections can be influenced by receiving accurate surgical and pharmacological treatment, with the potential to become future severity prognosis indexes.

## 1. Introduction

Odontogenic cervicofacial phlegmons are severe infections of the superficial and deep anatomical spaces of the head and neck. This specific pathologic state results from existing pathogenic bacteria that are localized primarily in the tooth structures or periodontal tissues. Odontogenic infections have the potential to enter the spongy bone and spread in the surrounding soft tissue through the cortical plate. Other causes that can influence the dissemination of these pathogens in the soft tissue can be represented by the postoperative status and complications of teeth exodontia, salivary gland infections, or malignancies of the oral structures [1]. The existent studies in the literature describe as a frequent complication associated with the lower impacted molars, namely the occurrence of infectious events with a high chance of disseminating in the nearby anatomical regions. Due to this aspect, the American National Institute of Health (NIH) suggests that impacted third molars should be removed in case they are the cause of any pathological changes [2].

One of the main characteristics of this type of infection is its potential to evolve and spread along the fascial planes of the neck from the skull base to the mediastinum, being potentially life-threatening, characterized by a fast evolution that requires immediate assessment due to the risk of airway blockage [3]. Potentially fatal complications include descending mediastinitis, internal jugular vein thrombosis, arterial erosion, pneumonia, meningitis, empyema, lung abscess, sepsis, and intracranial extensions, especially in patients who are immunocompromised or have comorbid conditions [4,5,6,7]. Any of these signs that accompany a cervicofacial infection must be seen as a warning by the clinician.

Typically, the normal flora of the oral cavity serves as causative organisms. The most involved bacteria species are represented by polymicrobial flora, such as *Streptococci*, *Peptostreptococcus*, *Staphylococcus aureus*, and anaerobes [8].

The diagnosis must be prompt and based on the classic symptoms: pain, fever, dysphonia, dyspnea, trismus, anterior floor edema, limitation of tongue protraction, oropharyngeal edema, and congestion of the cervicofacial skin [8,9].

It is important to treat this infection as an emergency situation, which requires to the upper airways to be secured, immediate surgical drainage of the infected spaces, removal of the source of the infection, and antibiotic treatment. Even with a rapid diagnosis and treatment, the evolution of some cases can be unfavorable. An explanation for this could be found in the host response towards infection and the humoral immunity status.

The immune response can be assessed by the modified cellular populations and processes, which are represented by the secretion of cytokines, monocytes, neutrophils, eosinophils, mast cells, basophils and macrophages, followed by the activation of the complement [10,11]. The entire process can be described by the occurrence of hyperemia due to a vasodilatation process, followed by the extravasation of leukocytes and plasma, which determines an increase in the permeability and the diapedesis process of the neutrophils. Afterwards, the mechanisms determine the fibrin wall formation and the phagocytosis of the bacteria, resulting in the deposition by the macrophages of the necrotic material [12].

A new paraclinical tool that assesses systemic inflammation and uses the patient’s inflammatory biomarkers, namely the systemic immune-inflammation index (SII), can now be used [13]. This index is calculated based on the following formula: (N × P)/L (N, P, and L represent neutrophils, platelet, and lymphocyte, respectively). The SII is a novel inflammatory biomarker that can evaluate the prognosis in a wide range of diseases, including solid malignant tumors [14,15], pulmonary embolism [16], and coronary artery disease [17].

Making a comparison with other leukocytes, neutrophil and lymphocyte values are crucial to the evolution of the infectious process. A typical reaction of the leucocytes is an increase in neutrophils. Compared to other WBC subpopulations, the neutrophil–lymphocyte ratio (NLR) has a higher sensitivity in detecting the expansion of a systemic infection [18]. 

Besides the NLR, other markers can predict the severity of the case and its evolution: leukocyte count, C-reactive protein level (CRP), white blood cell count (WBC), and its fractions (neutrophils, lymphocytes, and monocytes) [19].

Our study aims to evaluate the predictive markers for odontogenic cervicofacial phlegmons and their evolution in order to establish potential correlations between biomarkers and the severity of the cases. To the best of our knowledge, no studies have investigated markers such as the SII, CRP, and WBC levels, either alone or combined, before and after the surgical procedure and pharmaceutical treatment.

## 2. Materials and Methods

The present retrospective study is based on diagnosed cases of odontogenic cervicofacial phlegmons, admitted to the Maxillofacial Surgery Department of Emergency Hospital Timisoara between January 2012 and December 2022. The included data were collected from the medical charts of patients and were represented by age, gender, diagnosis of odontogenic cervicofacial phlegmons, duration of hospitalization, treatment and bloodwork values, and clinical and paraclinical examination outcomes. 

Our study was approved by the Ethics Committee of “Victor Babeș” University of Medicine and Pharmacy Timișoara (no. 09/2023), and patients agreed and signed an informed consent form that followed the guidelines of the Declaration of Helsinki.

### 2.1. Recruitment

In this study, we included patients based on the following inclusion and exclusion criteria.

Inclusion criteria:

- Age: 18–80 years
- Both males and females
- Diagnosis of odontogenic cervicofacial phlegmons, according to the ICD-10 disease classification
- Hospitalized patients in the Maxillofacial Surgery Department
- Patients with clear indication of surgical treatment

Exclusion criteria:

- Age: <18 years
- Incomplete medical charts
- Pregnancy
- Non-odontogenic head and neck infections

### 2.2. Blood Samples

The admission and examination of patients for the hospitalization process were performed according to the guidelines of the Maxillofacial Department. Antecubital venous blood was collected on day 1, i.e., admission, and on the last day in hospital, i.e., before discharge. For biomarker determination, a routine blood examination was performed immediately after the blood samples were collected. 

For the SII calculation, the following reference ranges were used: *neutrophil* counts (2.04–7.60 × 10^3^/μL); *platelet* counts (150–410 × 10^3^/μL); and *lymphocyte* counts (1.0–3.0 × 10^3^/μL). The formula used is as follows: SII = (neutrophil × platelet)/lymphocyte counts. The SII is expressed as ×10^3^/μL.

For the NLR calculation, the following reference ranges were used: *neutrophil* counts (2.04–7.60 × 10^3^/μL); *lymphocyte* counts (1.0–3.0 × 10^3^/μL). The NLR was measured by dividing the number of neutrophils by the number of lymphocytes. A normal range of NLR is between 1–2, while values higher than 3.0 and below 0.7 in adults are pathological [18].

The following blood test data were also investigated on admission: *CRP* (0–9 mg/L); *WBC* count (4.0–10.0 × 10^3^/μL); and *neutrophil*, *lymphocyte*, and *platelet* count (150.0–410.0 × 10^3^/μL).

All patients were treated with similar surgical approaches and antibiotic therapy. 

### 2.3. Patient Management

Odontogenic cervicofacial phlegmons infections were managed with a standard protocol. Anamnesis was the first step performed; afterwards, a clinical examination and anesthesiologist consultation were carried out. At the presentation in the clinic, all patients presented dysphagia, trismus, severe edema, tegument congestion, masticatory and deglutition alterations, at least one episode of fever, and a history of tooth pain. Preoperative images were performed, namely cervicofacial computerized tomography scan (CT scan) and dental panoramic X-ray. After all the information was provided by the clinical and paraclinical examinations, the diagnosis was found to be cervicofacial phlegmons of an odontogenic cause. 

The aseptic conditions required were applied and respected. The asepsis of the cervicofacial skin was performed with povidone Betadine solution 10% (Egis Pharmaceuticals, Budapest, Hungary). Oral-cavity aseptization was performed with Chlorhexidine 2% (Ana, Romania). Causal teeth were extracted in order to ensure the drainage of the cervicofacial spaces. At this time, sampling of the collection was performed. Bacteriological samples were cultured in aerobic and anaerobic conditions. Incision, drainage, and debridement were performed for all anatomic cervical fascial spaces involved in the infectious process. Collections were drained with intraoral, transcervical, or combined approaches. Drains were placed through the opened incisions and retained with a suture in order to realize large lavages with successive solutions: 0.9% saline (B. Braun Melsungen AG, Melsungen, Germany), Metronidazol Braun 5 mg/mL (B. Braun Melsungen AG); Betadine 100 mg/mL (Egis Pharmaceuticals). Every day, patients had clinical check-ups, irrigation with the combination of the aseptic solutions, and sterile dressing. 

All patients received intravenous probabilistic antibiotherapy effective against oral flora, namely Ceftriaxon 1 g- 2 × 1/day and Gentamicin 80 mg/2 mL- 2 × 1/day. After the bacteriologic exam, the antibiotics were adapted to bacteriological results. Postoperative CT scans were performed only in cases with poor evolution. 

From all the patients, before hospital discharge, an antecubital venous blood sample was collected in order to again determine the systemic immune-inflammation Index (SII); the neutrophil–lymphocyte ratio (NLR); WBC; CRP level; and neutrophil, lymphocyte, and platelet count. 

### 2.4. Statistical Analysis

Continuous data are presented as means with SD where data are normally distributed and as medians with the 25th and 75th centiles for non-parametric data. Categorical data are summarized as frequencies and percentages. Differences between groups for continuous normally distributed data were tested using Welch’s *t*-test for two groups or ANOVA when there were more than two groups. Non-parametric continuous data were tested using a Mann–Whitney U test for two groups or Kruskal–Wallis test for three or more groups. Differences across categorical data were tested using the χ^2^ test or Fisher’s exact test when expected cell counts were less than five. Continuous variable distributions were tested for normality using the Shapiro–Wilk test. All statistical analyses were carried out with R (version 3.6.3; R Foundation for Statistical Computing, Vienna, Austria) using the tidyverse, finalfit, mcgv, stringdist, janitor, and Hmisc packages.

## 3. Results

After the application of the inclusion and exclusion criteria, a total of 39 patients that were diagnosed with cervicofacial phlegmons were included in the study: 17 females (43.6%) and 22 males (56.4%). A total of 29 patients were excluded due to incomplete medical charts. The mean age of the subjects included in the present study was 47.7 years (SD = 18.08). After the analysis of the clinical and paraclinical aspects, in the case of all the patients included in the study, the causative odontogenic agent was the lower molars. The average calculated hospitalization period related to the included cases was 16.6 days (SD = 11.8). 

Regarding the course of care, 12 patients were directly addressed by the dentist (30.8%), 14 by the emergency department (35.9%), 4 (10.2%) by the general practitioner, and 9 (23.1%) addressed themselves. A number of 18 patients (46.2%) were consulted by a minimum of 2 different doctors before being referred to our department (general practitioner, dentist, or emergency doctor).

Before the hospital presentation, 25 patients (39%) received oral antibiotic therapy. The general practitioner, dentist, and emergency physician recommended the antibiotic treatment, or antibiotics were taken by self-medication. Treatment duration before presenting to the Oral and Maxillofacial Department was 5.4 days. The majority of the patients (14 patients; 56%) were on amoxicillin or amoxicillin and clavulanic acid antibiotic therapy, 5 patients (20%) took oxacillin, 4 patients (16%) took clindamycin, and 2 patients (8%) took ampicillin.

Regarding the anti-inflammatory treatment before presenting to the hospital, a total number of 26 patients (66%) had oral-administrated treatment. Out of them, 21 patients (80.8%) received nonsteroidal anti-inflammatory drugs (NSAIDs), and 5 patients (19.2%) received oral corticosteroids.

### 3.1. Systemic Immune-Inflammation Index (SII)

Regarding the SII, in Table 1, the values after the laboratory procession of the blood collected on day 1, i.e., admission to hospital, and the SII values after the surgical approach and pharmacological treatment of the cervicofacial phlegmons are presented (SIIa).

Our study investigated the changes in the systemic immune-inflammatory index (SII) associated with surgical intervention. Preoperatively, the patients exhibited a median SII value of 2006.1 within an interquartile range (IQR) of 1750.0 to 3261.5, signifying heightened systemic inflammation. Postoperative measurements revealed a notable reduction in the SII, with a median value of 1453.4 and an IQR of 813.7 to 2436.6. Across all observations, the SII median was 1833.9, encapsulated by an IQR of 997.6 to 2772.9. The significant drop in SII post-surgery, as indicated by a *p*-value of 0.023, underscores the efficacy of the surgical intervention in attenuating systemic immune-inflammatory responses. These data are represented in Table 1.

**Figure 1:**
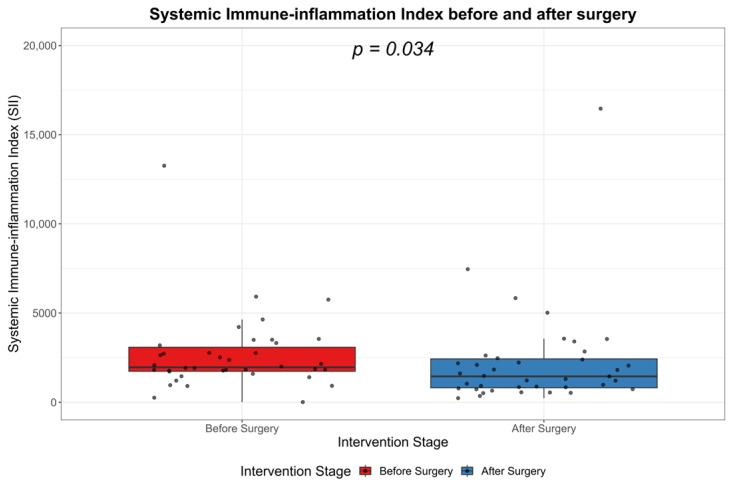
Boxplot showing the SII before treatment and SII after the surgical and pharmacological treatment.

The values of the SII significantly decreased after the surgical approach and pharmacological treatment of the cervicofacial phlegmons, as represented in Figure 1. 

### 3.2. Neutrophil–Lymphocyte Ratio (NLR)

The initial assessment revealed a high median NLR of 9.6, with the interquartile range (IQR) situated between 6.4 and 14.3, suggesting a substantial inflammatory response. However, postoperative evaluations recorded a significant decrease in the NLR, where the median value dropped to 4.4 and the IQR narrowed down to 3.6 to 7.3. The median NLR value for the entire data set was 6.5, with an IQR of 4.4 to 12.1. The considerable reduction in the NLR following surgery, as demonstrated by a *p*-value of less than 0.001, reinforces the positive impact of the surgical intervention in mitigating systemic inflammation, as Table 2 shows.

**Figure 2:**
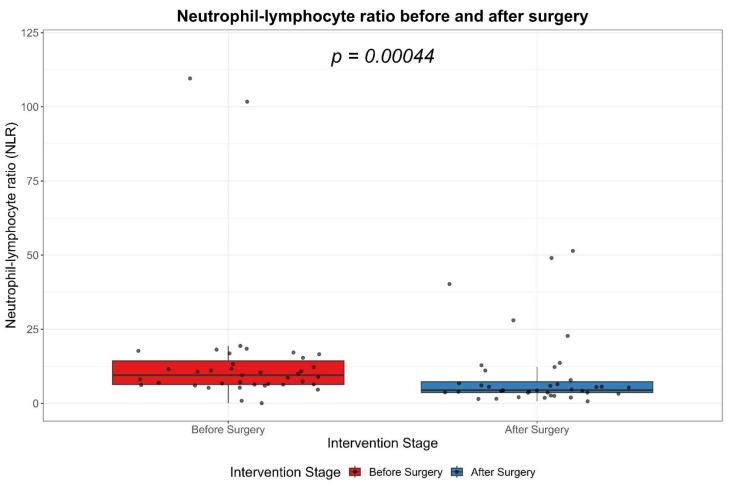
Boxplot showing the NLR levels before and after the treatment.

The values of the NLR significantly decreased after the surgical treatment of the cervicofacial phlegmons, as demonstrated by Wilcoxon signed-ranks test (Figure 2).

### 3.3. C-Reactive Protein Level (CRP)

C-reactive protein (CRP) levels were evaluated both before and after surgery. The median CRP level prior to surgery was considerably high at 133.6, with an interquartile range (IQR) of 55.5 to 228.4. Following surgical intervention, there was a significant reduction in CRP levels, with a median value of 2.4 and an IQR of 1.1 to 6.5. When considering all observations, the overall median CRP level was found to be 13.5, with an IQR of 2.5 to 208.0 (Table 3). The observed decrease in CRP levels post-surgery was statistically significant, with a *p*-value of less than 0.001. This suggests that the surgical intervention had a substantial impact on reducing systemic inflammation, as indicated by the CRP levels.

**Figure 3:**
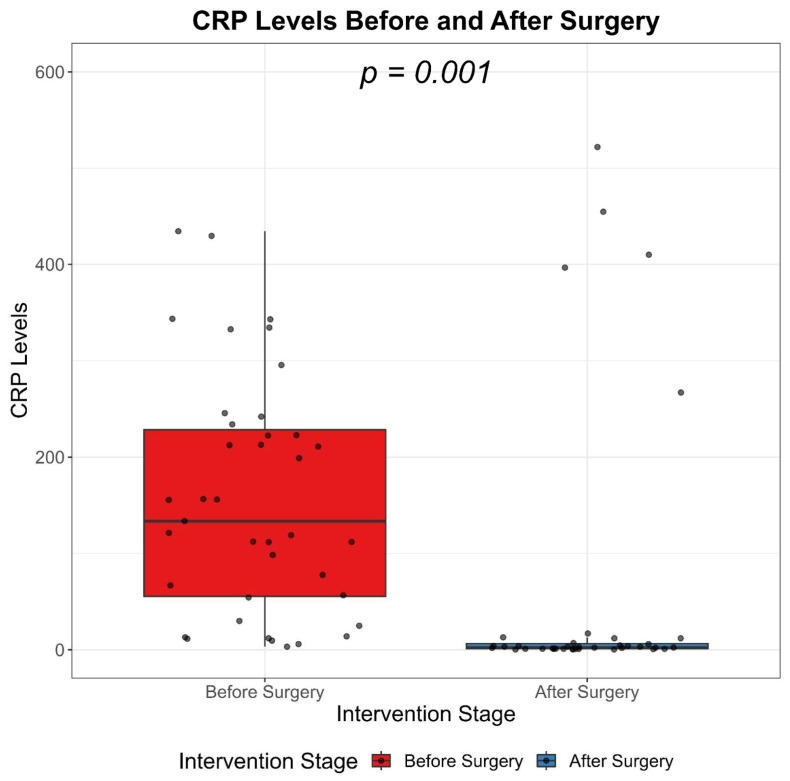
CRP-Mediated Inflammation Assessment: Before and After Surgical and Pharmacological Intervention.

The values of CRP significantly decrease after the surgical approach and pharmacological treatment of the cervicofacial phlegmons, demonstrated by Wilcoxon signed-ranks test (Figure 3).

### 3.4. WBC Levels

**Figure 4:**
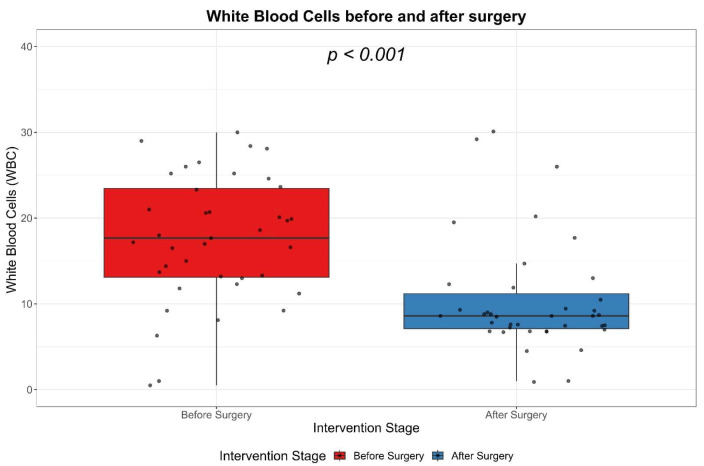
Changes in WBC Count: A Pre- and Post-Treatment Evaluation.

We examined the white blood cell (WBC) count both before and after surgery. Prior to surgery, the median WBC count was elevated at 17.7, with an interquartile range (IQR) of 13.1 to 23.5. Following surgical intervention, a significant decrease in the WBC count was observed, with a median value of 8.6 and an IQR of 7.1 to 11.2. Considering all data points, the overall median WBC count was 12.3, with an IQR of 7.9 to 19.8. The reduction in WBC count post-surgery was found to be statistically significant, with a *p*-value of less than 0.001 (Table 4). This indicates that the surgical procedure significantly influenced the reduction in systemic inflammation, as evidenced by the drop in WBC count (Figure 4).

## 4. Discussion

The odontogenic cervicofacial phlegmons represent a medical and surgical maxillofacial emergency that affect the superficial and deep anatomical spaces of the face and neck. It is estimated that incidence is around 10/100,000/year, with an ascending trend especially related to the age group under 5 years, where the reported incidence is around 2/100,000 cases/year [20]. Nevertheless, studies have shown the fact that these types of infections do not follow a specific age or gender trend. 

The major complications that can occur are represented by the progression of the infection to the mediastinum, necrotizing fasciitis of the cervical area, artery rupture, jugular vein thrombosis, empyema, pneumonia, or airway obstruction [8]. Applying efficient treatment can decrease the incidence of local and general complications and reduce mortality. 

Furthermore, studies report that a consequence of the severe periodontal disease and applied surgical treatment is dissemination and sepsis status by a direct spread into the bloodstream of the pathogenic microorganisms. In this case, oral antibiotic treatment may be an ineffective approach, suggesting the need for local antibiotic therapy, in most of the cases based on metronidazole [21]. A solution in these cases could be the use of local drug-delivering devices that can provide a high concentration of antibiotic directly where needed, avoiding and limiting the general side-effects of this type of medication.

Being a severe microbial infection of the cervicofacial spaces that originates from the periodontal spaces, a systemic inflammatory reaction from the host is initiated in order to eradicate the infection and stop the progression of general complications. This self-defense reaction manifests in general signs: fever, tachycardia, tachypnea, and hypotension. The loco-regional signs of inflammation are represented by cervicofacial skin alterations, namely the presence of the tenderness, tension, edematous, and a woody aspect of the area. Rubor, calor, tumor, dolor, and functio laesa are signs that can also be present. The lymphadenopathy of the sublingual, submental, or cervical nodes is often encountered in these cases [22,23].

Rapid management of these patients must be based on a series of serum markers that can evaluate and demonstrate the efficiency of the pharmacological and surgical treatment. This study aimed to determine the impact of different serum markers in the cases’ evolution, taking into consideration their single and combined value, on variables that were assessed at the admission in the hospital and on the discharge of the patients. 

We identified important changes that can be observed in the laboratory examination, such as alterations of the levels of WBC, C-reactive protein (CRP), lymphocytes, and platelet count. 

White blood cell count (WBC) and its fractions (neutrophils, lymphocytes, monocytes) are often used as objective evaluation parameters. However, their values alone cannot determine disease severity [13]. Our study confirms the literature findings that at hospital admission of the patients with cervicofacial phlegmons, the levels of WBCs are higher and with proper treatment, the WBC counts decreases [24]. In case of the patients included in our study, the WBC count significantly decreased after the treatment of the cervicofacial phlegmons, as demonstrated by Wilcoxon signed-ranks test (*p* < 0.001).

CRP is frequently measured on admission when odontogenic infections are suspected. CRP levels are objective and repeatable during the hospitalized period. Constant monitorization of CRP levels is an effective tool in the detection of sepsis, showing a higher sensitivity compared to white blood cell count or body temperature measurement. It was proven that this inflammatory biomarker has an increased level in the case of viral infections, bacterial implications, trauma, several systemic diseases, or in the case of post-surgical status [25]. The level of CRP is the highest after 48 h from the onset of the inflammatory response. Nevertheless, assessing strictly the levels of CRP in odontogenic infections and their relationship with the evolution of the patients cannot be yet considered a risk prediction tool [26,27,28].

In our study, the values of CRP significantly decreased after the surgical approach and pharmacological treatment of the cervicofacial phlegmons, reaching statistical significance (*p* < 0.001). 

A new and promising indicator that can predict the prognosis of patients with severe odontogenic infections is the systemic immune-inflammation index (SII). This index is calculated based on the following formula: (N × P)/L (N, P, and L represent neutrophil, platelet, and lymphocyte, respectively, from peripheral blood). In the literature, a few studies have investigated the SII index in odontogenic cervicofacial phlegmons. Furthermore, in the case of deep odontogenic infections, its applicability has not yet been clearly established [29]. The values of the SII in our study significantly decreased after the surgical approach and pharmacological treatment of the cervicofacial phlegmons (*p* = 0.012). From this hypothesis, we conclude that we can use this index both for initial evaluation of the cervicofacial phlegmons and to assess the treatment response.

The host response to a bacterial infection is due to the activation of neutrophils that are implicated in the phagocytosis process, neutralizing the bacteria. Studies reveal the fact that NLR levels increase in patients diagnosed with deep neck infections, suggesting its potential as a predictor for bacteremia. Assessment of these levels has the potential to be a simple, easy, and cost-effective test for the quantification of the inflammatory status and complication risk in the case of neck infections [30].

Our results support the notion that the NLR values are higher at hospital admission of patients with cervicofacial phlegmons. In our study, the NLR decreased significantly after the surgical treatment of cervicofacial phlegmons.

The diagnosis and treatment of the cervicofacial phlegmons must be initiated as soon as possible due to the complications and higher costs that appear when the disease progresses. 

In the study performed by Saluja et al. [31], their results report that in terms of treatment items and costs, the most expensive were the costs provided by the operative management (USD 4379/OR trip), followed by the costs in ICU management (USD 3541/day) and the PICC line placement (over USD 2000). In case of the management of head and neck infections, the costs related to the treatment of abscesses were reported to be the highest (USD 22,384), followed by the treatment of phlegmons cases (USD 18,969) and cellulitis (USD 10,490). The reports related to the average cost of treatment received reveal that in the case of the surgical management of cases, the cost was approximately USD 22,071, exceeding the costs related to the use of IV antibiotics alone (USD 14,950) [31]. 

In the case of phlegmons, antibiotic therapy must be installed even before the microbial culture report. Usually, the first choice is the administration of amoxicillin and clavulanic acid, targeting the microorganisms that are encountered in the oral environment. Taking into consideration the life-threating potential of these infections, several researchers suggest the use of a combination of penicillin with a -lactamase inhibitor, or an a-lactamase inhibitor associated with another medication (clindamycin or metronidazole) with a higher effect against anaerobes [32,33]. Our study supports the literature recommendations. 

Odontogenic infections that progress towards cervicofacial phlegmons represent a life-threatening complication that requires attention from clinicians, an adequate performance of the clinical examination, and interpretation of the paraclinical aspects. By taking into consideration, during diagnosis and prognosis evaluation, the implications of the values of inflammatory markers, further treatment approaches can be carried out according to the severity of the encountered changes. A correct evaluation of the case and proper surgical and pharmaceutical treatment can positively influence the inflammatory parameters and immune response.

Regarding the limitations of our study, we can include the fact that it was a single-center study with a small number of patients that were hospitalized with cervicofacial phlegmons. Nevertheless, the results of this study offer a new perspective and could be further used as a basis for a larger multicentric study.

## 5. Conclusions

The immune response and hematological and inflammatory changes related to severe infections can represent a starting point for the identification of potential blood values to be used as adjunctive tools for the diagnosis and evolutive prognosis of these infections.

Cervicofacial odontogenic infections are characterized by an aggressive and unpredictable evolution, and our study outlined the dynamic changes in biomarkers when accurate surgical and pharmacological treatment was performed, influencing the final outcome of the cases.

## Figures and Tables

**Table 1:** Assessment of Systemic Immune-Inflammatory Index (SII) at Admission and Post-Treatment in Hospitalized Patients (Wilcoxon Signed-Ranks Test).

Variable	Before Surgery	After Surgery	Total	*p*
SII	2006.1 (1750.0 to 3261.5)	1453.4 (813.7 to 2436.6)	1833.9 (997.6 to 2772.9)	0.023

**Table 2:** Assessment of *Neutrophil–lymphocyte ratio (NLR)* at Admission and Post-Treatment in Hospitalized Patients.

Variable	Before Surgery	After Surgery	Total	*p*
NLR	9.6 (6.4 to 14.3)	4.4 (3.6 to 7.3)	6.5 (4.4 to 12.1)	<0.001

**Table 3:** Assessment of *CRP levels* at Admission and Post-Treatment in Hospitalized Patients.

Variable	Before Surgery	After Surgery	Total	*p*
CRP	133.6 (55.5 to 228.4)	2.4 (1.1 to 6.5)	13.5 (2.5 to 208.0)	<0.001

**Table 4:** Assessment of *WBC Levels* at Admission and Post-Treatment in Hospitalized Patients.

Variable	Before Surgery	After Surgery	Total	*p*
WBC	17.7 (13.1 to 23.5)	8.6 (7.1 to 11.2)	12.3 (7.9 to 19.8)	<0.001

## Data Availability

Data supporting reported results can be provided on request.
